# Retraction Note: Integrin-β5, a miR-185-targeted gene, promotes hepatocellular carcinoma tumorigenesis by regulating β-catenin stability

**DOI:** 10.1186/s13046-023-02762-6

**Published:** 2023-07-19

**Authors:** Zhikun Lin, Ruiping He, Haifeng Luo, Chang Lu, Zhen Ning, Yuanhang Wu, Chuanchun Han, Guang Tan, Zhongyu Wang

**Affiliations:** 1 Department of Hepatobiliary Surgery of the First Affiliated Hospital& Institute of Cancer Stem Cell, Dalian Medical University, Dalian, 116027 China; 2 Department of Oncology of the First Affiliated Hospital, Dalian Medical University, Dalian, 116027 China

**Retraction Note:** ***J Exp Clin Cancer Res*** **37, 17 (2018)**

10.1186/s13046-018-0691-9

The Editor-in-Chief has retracted this article. After publication, concerns were raised regarding highly similar images in the figures. Specifically:

- Figure 1b shRNA CTR MHCC-97L image appears highly similar to Fig. 6g shRNA CTR miR-185 inhibitor.
- Figure 3a MHCC-97L ITGB5 blot image appears highly similar to Fig. 3n WB: anti-Ub (stretched vertically).
- Figure 4h shRNA ITGB5 CTR image appears highly similar to Fig. 6g shRNA ITGB5 miR-185 inhibitor.
- Figure 4j shRNA ITGB5 and Fig. 6i shRNA CRT miR-185 inhibitor images appear to overlap.
- Figure 6j shRNA β-catenin CTR and shRNA ITGB5 miR-185 inhibitor images appear to overlap.

The authors have stated that the images were misused, and provided the raw data for validation. However, due to the number and severity of concerns, the Editor-in-Chief no longer has confidence in the presented data.

Zhikun Lin has stated on behalf of all co-authors they do not agree to this retraction.

